# AGImpute: imputation of scRNA-seq data based on a hybrid GAN with dropouts identification

**DOI:** 10.1093/bioinformatics/btae068

**Published:** 2024-02-05

**Authors:** Xiaoshu Zhu, Shuang Meng, Gaoshi Li, Jianxin Wang, Xiaoqing Peng

**Affiliations:** School of Computer and Information Security, Guangxi Key Laboratory of Trusted Software, Guilin University of Electronic Technology, Guilin 541004, China; School of Computer Science and Engineering, Guangxi Normal University, Guilin 541006, China; School of Computer Science and Engineering, Guangxi Normal University, Guilin 541006, China; School of Computer Science and Engineering, Hunan Provincial Key Lab on Bioinformatics, Central South University, Changsha 400083, China; School of Life Sciences, Center for Medical Genetics, Central South University, Changsha 400083, China

## Abstract

**Motivation:**

Dropout events bring challenges in analyzing single-cell RNA sequencing data as they introduce noise and distort the true distributions of gene expression profiles. Recent studies focus on estimating dropout probability and imputing dropout events by leveraging information from similar cells or genes. However, the number of dropout events differs in different cells, due to the complex factors, such as different sequencing protocols, cell types, and batch effects. The dropout event differences are not fully considered in assessing the similarities between cells and genes, which compromises the reliability of downstream analysis.

**Results:**

This work proposes a hybrid Generative Adversarial Network with dropouts identification to impute single-cell RNA sequencing data, named AGImpute. First, the numbers of dropout events in different cells in scRNA-seq data are differentially estimated by using a dynamic threshold estimation strategy. Next, the identified dropout events are imputed by a hybrid deep learning model, combining Autoencoder with a Generative Adversarial Network. To validate the efficiency of the AGImpute, it is compared with seven state-of-the-art dropout imputation methods on two simulated datasets and seven real single-cell RNA sequencing datasets. The results show that AGImpute imputes the least number of dropout events than other methods. Moreover, AGImpute enhances the performance of downstream analysis, including clustering performance, identifying cell-specific marker genes, and inferring trajectory in the time-course dataset.

**Availability and implementation:**

The source code can be obtained from https://github.com/xszhu-lab/AGImpute.

## 1 Introduction 

Single-cell RNA sequencing (scRNA-seq) data provide gene expression profiles at a single-cell resolution which can help biological researchers identify cell types and explore the process of cell development (Lee *et al.*, 2022, Anderson *et al.*, 2023, Song *et al.*, 2023). However, scRNA-seq data are characterized by high sparsity and high noise, with 65%–90% zeros including true and false zero counts (Gan *et al.*, 2022). True zeros indicate that the genes are not expressed, while false zeros are dropout events caused by technical noise (Grün *et al.*, 2014), such as low capture efficiency of mRNA, amplification bias, and library size differences. The numerous dropout events will compromise the reliability of the downstream analysis of scRNA-seq data. Therefore, identifying and imputing dropout events are important tasks for improving the quality of scRNA-seq data (Vallejos *et al.*, 2017, Raevskiy *et al.*, 2023).

In recent years, two groups of dropout event imputation methods for scRNA-seq data have been proposed. One class is to re-estimate all gene expression values while imputing dropout events, and the other is to identify the dropout events from the zeros first and then impute them. In the first category, several statistical model-based dropout imputation methods have been developed by borrowing information from similar cells or genes, which commonly involve clustering cells or genes and then imputing dropout events (Berrevoets *et al.*, 2023, Mahmoudi, 2023). SAVER (Huang *et al.*, 2018) borrowed information from similar genes, then used the average of output posterior distribution as the new predicted gene express values. MAGIC (van Dijk *et al.*, 2018) utilized Markov transfer matrices to estimate all gene expression values in similar cells, while VIPER (Chen and Zhou, 2018) borrowed information from similar cells within cell subtypes identified by regression. ENHANCE (Wagner *et al.*, 2019) borrowed information from neighboring cells and then estimated all expression values of low-noise principal components (PC) genes. These genes were determined based on the evaluation of technical noise captured by a single PC. In addition, some deep learning-based methods have been proposed to integrate gene expression distribution for imputation. For example, scIGANs (Xu *et al.*, 2020) utilized a Generative Adversarial Network (GAN) to generate new values as imputations for dropout events but facing the challenge of losing biological information due to a potential mismatch between the generated data and the original scRNA-seq data. The above-mentioned methods change most gene expression values, which lead to excessive imputation and potentially introduce new noise into scRNA-seq data.

To address the excessive imputation, some statistical model-based methods have been developed that involve identifying dropout events before imputation. For example, scImpute (Li and Li, 2018) identified dropout events through estimating the dropout probability using a statistical model and imputed the identified dropouts subsequently. scLRTC (Pan *et al.*, 2021) used cell similarity measurements and constructed a third-order tensor to estimate the gene expression values, in which some zero values were re-estimated and nonzero values were kept. These changed zeros were identified as dropout events, and their estimated values were taken as the dropout imputations. However, the number of true zeros varies among different cells due to cell heterogeneity and batch effects, which is ignored in these studies. Differentially estimating the number of dropout events for different cells is critical for effectively imputing dropout events.

Hence, we have proposed a method, named Autoencoder-GAN based imputation for scRNA-seq data (AGImpute). In AGImpute, a dynamic threshold estimation mechanism is designed to adaptively identify the number of dropout events for different cells. Then, an Autoencoder-GAN model is constructed to impute the identified dropout events by leveraging information from both similar cells and gene expression distributions. To evaluate its performance, AGImpute is compared to seven existing dropout events imputation methods on two simulated and seven scRNA-seq datasets, based on several criteria, including the number of dropout imputations, clustering performance (Buettner *et al.*, 2015), cell-specific marker genes identification, and trajectory inference.

## 2 Materials and methods

**Figure 1.**
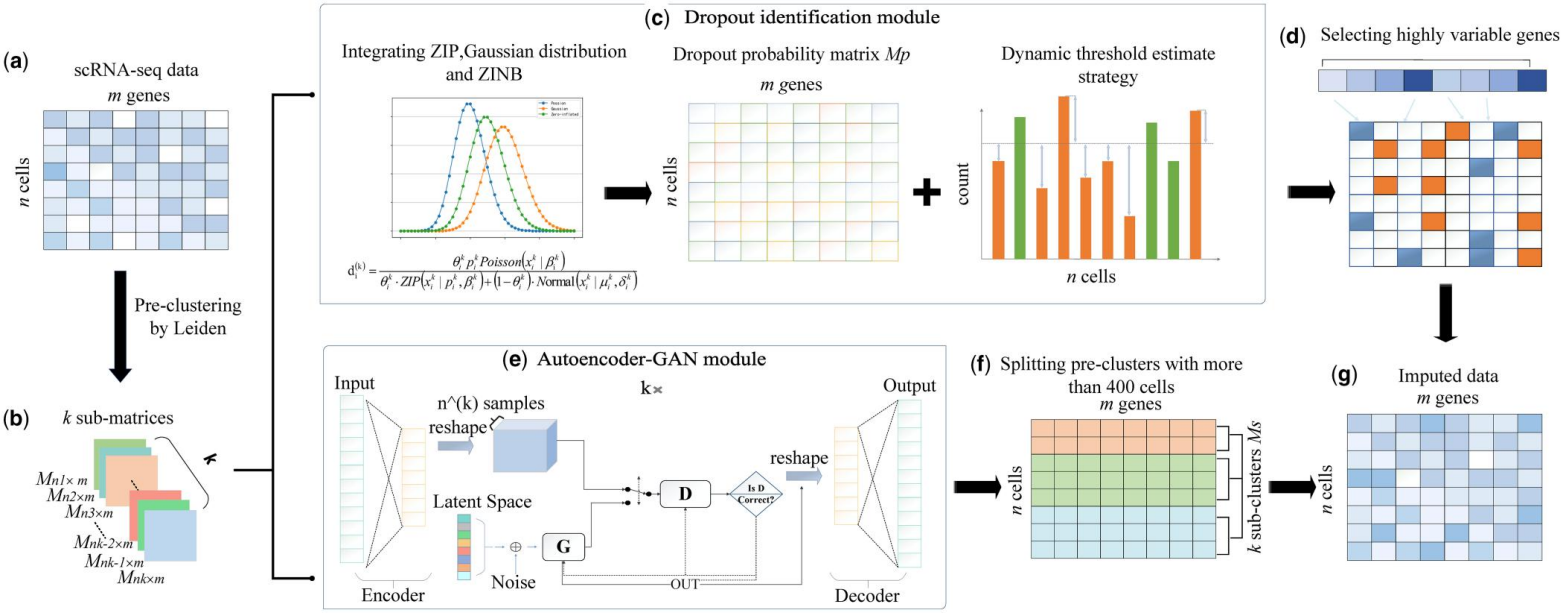
The workflow of AGImpute. (a) The input of gene expression matrix *M*. (b) Preclustering of matrix *M* into *k* submatrices using Leiden. (c) Design of dropout identification module with dropout probability matrix $M_{p}$ and dynamic threshold estimation. (d) Selection of final identified dropout events as an intersection of the top 4000 highly variable genes and identified dropout events. (e) Construction of Autoencoder-GAN module to reconstruct *k* submatrices and generate merged matrix $M_{g}$. (f) Smoothing of identified dropout events by splitting preclusters with more than 400 cells into subclusters $M_{s}$ by *k*-means, and calculation of mean for each subcluster. (g) Generation of an imputed matrix as output.

In this article, we consider that different cells have different numbers of dropout events, and propose AGImpute method containing a dropout identification module and an Autoencoder-GAN module, shown in Fig. 1. In the dropout identification module, a dropout probability matrix is constructed by defining a probability mass function (PMF), which fits scRNA-seq data based on the combination of Zero-inflated Poisson (ZIP), Gaussian, and Zero-inflated Negative Binomial (ZINB) distributions. Based on the dropout probability matrix, a dropout probability difference matrix is constructed, and a dynamic threshold estimation is proposed to determine the number of dropout events for different cells. In the Autoencoder-GAN module, the identified dropout events are imputed by combing Autoencoder (Fanai and Abbasimehr, 2023) and GAN (Goodfellow *et al.*, 2014) that incorporates preclustering labels from Leiden clustering.

### 2.1 Preclustering by Leiden

To incorporate information from similar cells, we perform Leiden clustering to group cells. The input gene expression matrix *M* contains *n* cells and *m* genes, where $m_{i,j}$ represents the expression of the *j*-th gene in the *i*-th cell. *n* cells are clustered into *k* clusters by Leiden, and the matrix *M* is divided into *k* submatrices: $M_{1\left(n_{1}\times m\right)},M_{2\left(n_{2}\times m\right)},\ldots ,M_{k\left(n_{k}\times m\right)}$.

### 2.2 Adaptive estimation of dropout events for different cells

A dropout identification module is used to identify dropout events in scRNA-seq data. In this module, a mixed distribution combining ZIP, Gaussian, and ZINB is used to fit scRNA-seq data and estimated dropout probability. Then, a dropout probability difference matrix is derived from the dropout probability matrix, and a dynamic threshold estimation is designed to adaptively predict the number of dropout events for different cells.

#### 2.2.1 Prediction of dropout probability based on a mixed distribution

Previous studies have used Poisson and ZINB distribution to fit scRNA-seq data, since the former can fit technical noise well and the latter can solve large amounts of zeros (Qiu *et al.*, 2023). To solve the problem of ZINB being unsuitable for high-noise data (Wang *et al.*, 2018), we design a mixed distribution combining ZIP, Gaussian, and ZINB to fit scRNA-seq data. In this mixed distribution, ZIP is used to fit the distribution of zeros including true and false zeros, and Gaussian distribution is used to fit the distribution of nonzeros. The probability statistical model is extremely sensitive to the initialization parameters, and ZINB can accurately capture the true and false zero ratio. So ZINB is used to initialize the parameters of ZIP and the proportion of dropout events. The PMF of ZIP is defined as in formula (1).

(1) fZIPY=y;ϕ,λ=ϕ+1-ϕe-λ,y=01-ϕe-λλyy!,y>0, 

where ϕ is the proportion of true zeros for a gene that is zero-expressed across all cells, and *y* is an indicator function that describes the number of expected false zeros. λ is the mean of the Poisson distribution.

The PMF of ZINB is defined as in formula (2).

(2) fZINBY=y;ϕ,λ,θ=ϕ + 1-ϕθθλ+θθ,y=01-ϕΓθ+yΓθy!λyθθλ+θθ+y,y>0 ,

where θ is the probability parameter of the Negative Binomial distribution.

The PMF of the Gaussian distribution is denoted as in formula (3).

(3) $$f_{\mathrm{Normal}}\left(x|\mu ,\sigma^{2}\right)=\frac{1}{\sqrt{2\pi }\sigma }e\mathrm{xp}\left(-\frac{{\left(x-\mu \right)}^{2}}{2\sigma^{2}}\right),\;$$

where μ represents the mean of all nonzeros and σ is the variance of all nonzeros.

Based on the three distributions, we define the final mixed distribution as in formula (4). To initialize the parameters, we perform the Expectation Maximization (EM) algorithm that would effectively estimate the parameters of a complex statistical model by iteratively optimizing the likelihood function.

(4) $$f_{x_{i}^{k}}\left(x\right)=\theta_{i}\cdot f_{\mathrm{ZIP}}\left(\phi_{i}^{k},\lambda_{i}^{k}\right)+\left(1-\theta_{i}\right)\cdot f_{\mathrm{Normal}}\left(\mu_{i}^{k},\sigma_{i}^{2k}\right),\;$$

$$\phi_{i}^{k},\lambda_{i}^{k}∼f_{\mathrm{ZINB}}\left(\phi^{k},\lambda^{k},\theta^{k}\right),$$

where *k* is the preclusters label, and $x_{i}^{k}$ is the expression of the *j*-th gene in the *i*-th cell belonging to the *k*-th cell cluster. $\theta_{i}$ is the proportion of zeros in all values. ${\;\phi }_{i}^{k}$, $\lambda_{i}^{k}$, $\mu_{i}^{k}$, and $\sigma_{i}^{2k}$ also denote the corresponding values in the *k*-th cell cluster, respectively.

Then, based on the final mixed distribution, the dropout probability of the *j*-th gene in the *k*-th cluster $d_{i}^{k}$ is defined as in formula (5). Finally, a dropout probability matrix $M_{p}$ is constructed by merging the *k* dropout probability submatrices.

(5) $$d_{i}^{k}=\frac{\theta_{i}^{k}\cdot p_{i}^{k}\cdot \mathrm{Poisson}\left(\lambda_{i}^{k}\right)}{\theta_{i}^{k}\cdot f_{\mathrm{ZIP}}\left(\phi_{i}^{k},\lambda_{i}^{k}\right)+\left(1-\theta_{i}^{k}\right)\cdot f_{\mathrm{Normal}}\left(\mu_{i}^{k},\sigma_{i}^{2k}\right)}.\;$$

#### 2.2.2 Adaptive estimation of the number of dropout events based on a dynamic threshold estimation mechanism

The number of dropout events is commonly set as a uniform threshold in all cells, failing to account for the differences between individual cells. To solve this problem, we develop a dynamic threshold estimation mechanism. Inspired by scRecover method (Miao *et al.*, 2019), we assume that adjacent genes with considerable variation in their dropout probabilities would be an indication of dropout events and try to adaptively estimate the dropout events by capturing these signals. First, genes are sorted in ascending order for every cell based on the dropout probability in the matrix $M_{p}$, resulting in a row-sorted matrix ${M\prime }_{p}$. Then, for each row in matrix ${M\prime }_{p}$, the differences between the dropout probabilities of adjacent elements (genes) are calculated, and a dropout probability difference matrix $M_{d}$ is defined as in formula (6).

(6) $$M_{d}=\left[\begin{array}{ccc} M_{p\left(0,1\right)}-M_{p\left(0,0\right)} & \cdots & M_{p\left(0,j\right)}-M_{p\left(0,j-1\right)}\\ \vdots & \ddots & \vdots \\ M_{p\left(i,1\right)}-M_{p\left(i,0\right)} & \cdots & M_{p\left(i,j\right)}-M_{p\left(i,j-1\right)} \end{array}\right].\;$$

Where $M_{p\left(i,j\right)}$ denotes the dropout probability of the *j*-th gene in the *i*-th cell.

By considering the maximum dropout probability difference and the number of corresponding dropout events, we define the threshold of the number of dropout events for different cells. For the *i*-th cell in the matrix $M_{d}$, the maximum dropout probability difference is the maximum value in the *i*-th row. For *n* cells, let $V_{\max}=\left[\max\left\{M_{d}\left(0,.\right)\right\},\ldots ,\max\left\{M_{d}\left(i,.\right)\right\},\ldots ,\max\left\{M_{d}\left(n-1,.\right)\right\}\right]$, where $\max\left\{M_{d}\left(i,.\right)\right\}$ denotes the maximum value in the *i*-th row. Let $V_{\mathrm{num}}=\left[N_{0},\ldots ,N_{i},\ldots ,N_{n-1}\right]$, where $N_{i}$ denote the vector of the number of elements in each row *i* with an index less than *j*, supposing the *j*-th element in the *i*-th row corresponds to $\max\left\{M_{d}\left(i,.\right)\right\}$. Then, the top *t* maximum values in $V_{\max}\;$values are retained, and the mean of the corresponding values in $V_{\mathrm{num}}\;$is calculated, denoted as $m_{t}$. $m_{t}$ is the threshold for determining the number of dropout events.

To estimate the number of dropout events for each cell precisely, a regression strategy is developed to adjust $V_{\mathrm{num}}\left[i\right]$ dynamically. First, the difference between $V_{\mathrm{num}}\left[i\right]$ and $m_{t}$ is calculated. Then, $V_{\mathrm{num}}\left[i\right]$ is adjusted to approach $m_{t}$ by increasing it if it is less than $m_{t}$ or decreasing it otherwise. So, an updated ${V\prime }_{\mathrm{num}}\left[i\right]$ value is achieved, defined in formula (7). Based on the updated ${V\prime }_{\mathrm{num}}\left[i\right]$, the corresponding dropout probability is calculated for cell *i* in the dropout probability matrix $M_{p}$, and the dropout events are also identified in the gene expression matrix *M*, separately.

(7) V′numi=Vnumi+mtmt-VnumiVnumiω,mt≤VnumiVnumi+Vnumimt-Vnumimtω,mt>Vnumi, 

where ω is a regulation coefficient with an initial value of 1.

For a scRNA-seq dataset, we select the top 4000 high variable genes by using Scanpy, and the identified dropout events of these genes in each cell will be imputed.

### 2.3 Imputation for dropout events based on a hybrid Autoencod-er-GAN

A hybrid Autoencoder-GAN module is constructed to impute the identified dropout events. To consider the gene expression distribution, Autoencoder is used to learn a low-dimensional embedding space and GAN is used to generate the dropout imputation. To consider the similar cells, the *k* submatrices $M_{1\left(n_{1}\times m\right)},M_{2\left(n_{2}\times m\right)},\ldots ,M_{k\left(n_{k}\times m\right)}$ clustered by Leidon are input respectively, and *k* Autoencoder-GAN is obtained.

#### 2.3.1 Network architecture

The Autoencoder-GAN comprises an encoder, GAN, and decoder. In the encoder, there are six fully connected layers with 128 and 64 neurons, while the opposite layer structure is in the decoder. The GAN layer is embedded between the encoder and the decoder, including a generator and discriminator. The generator network uses Convolutional Neural Network (CNN) architecture with convolution kernels of (32,32,3), (32,32,3), (40,32,3), and (32,1,3). The discriminator network uses fully connected CNN layers with convolution kernels of (24,16,3,1,1), (16,8,3,1,1), (8,4,3,1,1), (4,4,3,1,1), (4,4,3,1,1), (4,4,3,1,1), and (4,1,2,1,0). The generator uses random noise as input and maps it to generated data with a similar distribution, while the discriminator judges whether the generated data are similar to the input data. After adversarial training, the GAN layer generates the dropout imputation.

#### 2.3.2 Training strategy

A mean squared error (MSE) loss function is used to minimize the difference between the input data of the encoder and the reconstructed data of the decoder, as defined in formula (8).

(8) $$\mathrm{MSE}=\frac{1}{n}\sum_{i=1}^{n}{\left(Y_{i}-{\hat{Y}}_{i}\right)}^{2},$$

where *n* is the number of samples.

In the GAN layer, the gene expression vector of each cell is transformed into a 100 × 100 matrix as input. Then, the generator generates synthetic data similar to the true data and the discriminator distinguishes between generated and true data. Adversarial training is performed using a mini-max objective function shown in formula (9), and stops after 200 epochs. Finally, the output matrix of the GAN is reshaped back into the gene expression matrix of cells. The *k* matrices reconstructed by the Autoencoder-GAN are combined into a new matrix.

(9) minGmaxDVD,G=Ex∼pdataxlog⁡Dx+Ez∼pzzlog⁡1-DGz,

where the random variable *x* follows the distribution $P_{\mathrm{data}}$of the true data, and the random variable *z* follows the distribution $p_{z}$ of the generated data.

#### 2.3.3 Imputation for dropout events

By considering the more similar cells, the *k*-means method is used to split preclusters larger than 400 cells into subclusters $M_{s}$. In each subcluster, a smooth interpolation is implemented to impute dropout events, that is, the dropout events are imputed by calculating the mean gene expression value across all cells.

### 2.4 Datasets

To test the ability of the AGImpute to impute dropout events, we applied AGImpute and other methods in two simulated datasets and seven scRNA-seq datasets. Two simulated datasets are generated by the Splatter R Package (Zappia *et al.*, 2017), including dropout data with a 60% dropout rate and the corresponding true data with a 0% dropout rate, both of them contained 20 000 genes, 1000 cells, and five cell types. The seven scRNA-seq datasets are downloaded from NCBI Gene Expression Omnibus (GEO) and summarized in Table 1.

**Table 1.** The list of seven real scRNA-seq datasets.

Accessed ID	Datasets	Platform	Tissue	#Cell type	#Cell	#Gene	Reference
GSE45719	Deng	Hiseq	Mus musculus	10	259	22 147	Deng *et al.* (2014)
GSE94333	Adam	Hiseq	Mus musculus	8	3660	23 797	Adam *et al.* (2017)
GSE81547	Enge	NextSeq	Human pancreas	6	2282	23 366	Chen *et al.* (2020)
GSE102299	Wallrapp	NextSeq	Mus musculus	3	752	45 686	Wallrapp *et al.* (2017)
GSE92332	Haber	10X	Mus musculus	9	1522	20 108	Haber *et al.* (2017)
GSE75748	CellType	Hiseq	Human Embryonic	2	1018	19 097	Chu *et al.* (2016)
GSE75748	Time-course	Hiseq	Human Embryonic	5	758	19 189	Chu *et al.* (2016)

## 3 Results

AGImpute is compared to seven state-of-the-art imputation methods, including ENHANCE, MAGIC, SAVER, scImpute, scIGANs, scLRTC, and VIPER. We first test the influence of selecting different numbers of highly variable genes in AGImpute, relating to the number of imputations. Then, we compare the performance of downstream analysis of the eight imputation methods, including clustering performance, identifying cell-specific marker genes, and inferring trajectory.

### 3.1 Analyzing the influence of selecting different numbers of highly variable genes

**Figure 2.**
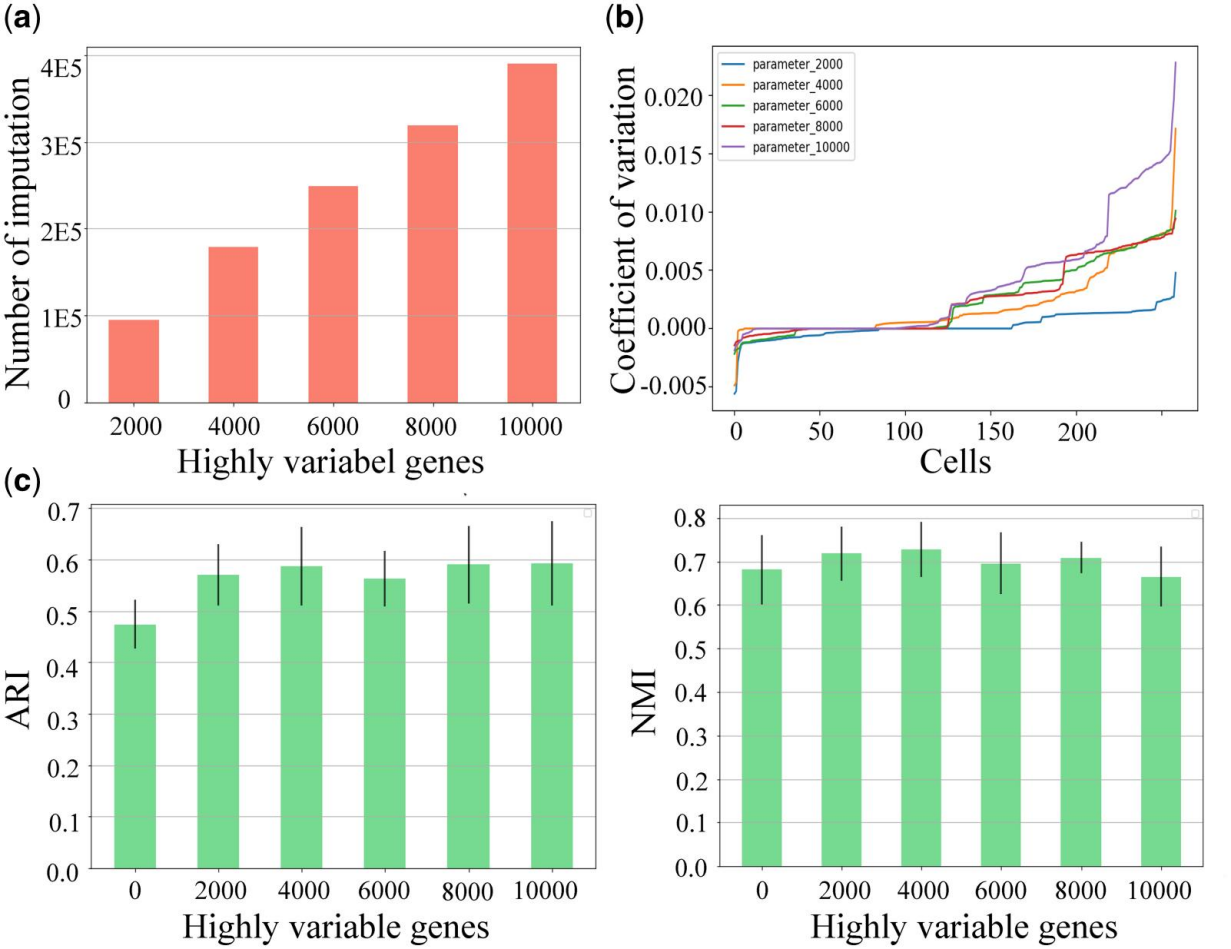
Influence of highly variable gene on dropout imputation in AGImpute. (a) Histogram of the number of dropout imputations. (b) Coefficient of variation between the raw data and the AGImpute imputed data. (c) Boxplots of Adjusted Rand Index and Normalized Mutual Information of clustering results on AGImpute-imputed data.

By selecting different numbers of highly variable genes in the Deng dataset, specifically 2000, 4000, 6000, 8000, and 10 000, we test the differences in the number of dropout imputations, coefficient of variation (CV), and clustering performance, shown in Fig. 2.

From Fig. 2a, the number of dropout imputations increases linearly with the number of highly variable genes. The number of dropout imputations with 10 000 highly variable genes is 4 times higher than that with 2000 highly variable genes. Figure 2b provides the CV between the raw data and the AGImpute-imputed data with different numbers of highly variable genes. From Fig. 2b, there are minimal dispersion differences before and after imputation for the first 125 cells, and gradual differences for the latter half. When the number of highly variable genes is 2000 or 4000, CV is more stable. Figure 2c presents the Adjusted Rand Index (ARI) and Normalized Mutual Information (NMI) of clustering results. It shows that there is a minimal influence on clustering performance for different numbers of dropout imputations. The best NMI and ARI are obtained when the number of highly variable genes is 4000. Therefore, we select the first 4000 highly variable genes to minimize the number of dropout imputations.

### 3.2 Comparing the similarity between the imputed data using different imputation methods and the raw data

**Figure 3.**
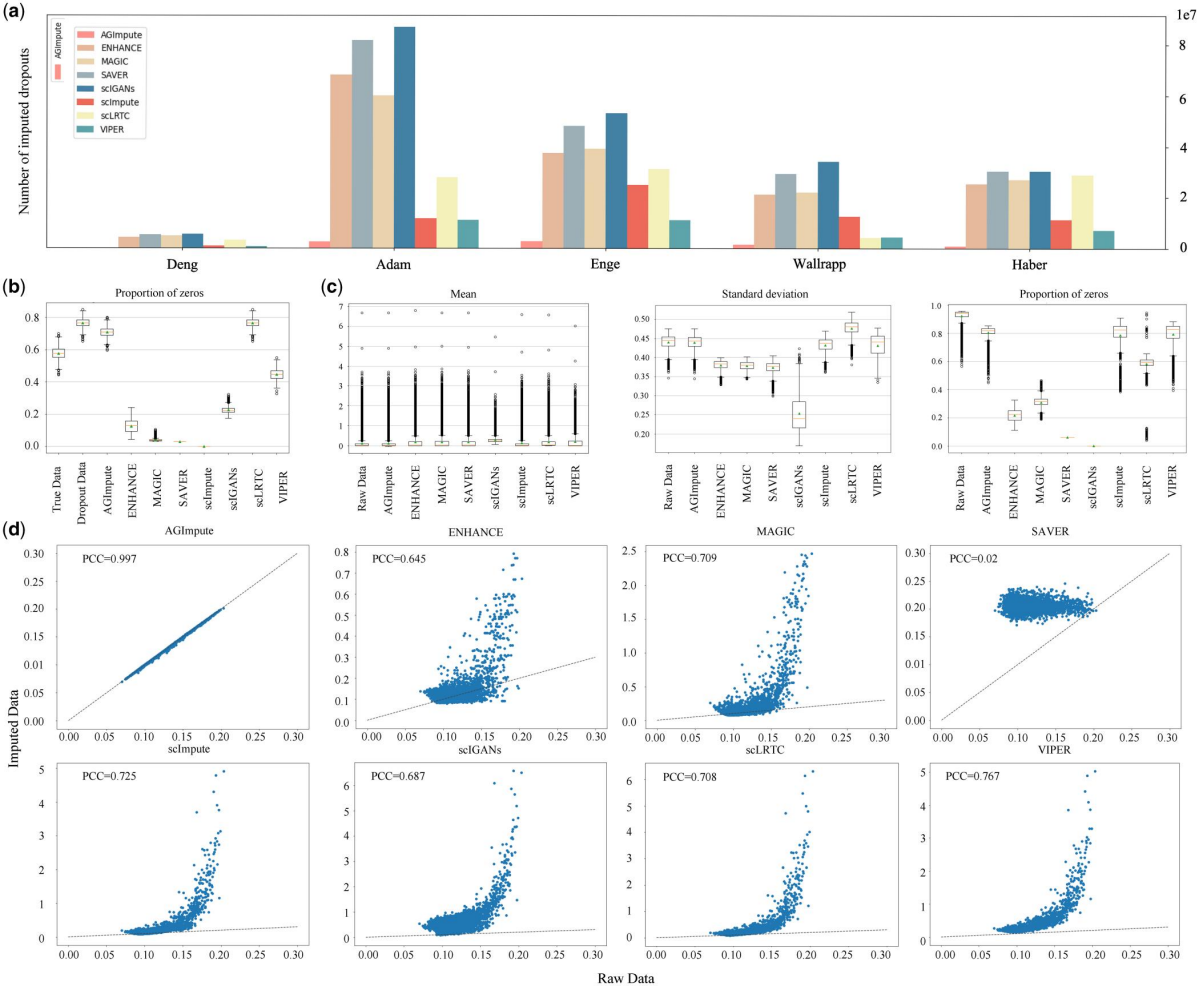
Comparison of imputation performance between AGImpute and other methods. (a) Histogram of the number of dropout imputed by different imputation methods. (b) Boxplots of the proportion of zeros in true data, dropout data, and imputed data on the simulated dataset. (c) Boxplots of the proportion of zeros, mean, and standard deviation of gene expression values on the real Adam dataset. (d) Gene expression density plot using eight interpolation methods on the Adam dataset, demonstrating differences between imputed and raw data.

To evaluate the ability to impute dropout events without excessive imputation, AGImpute is compared to seven imputation methods in terms of the number of dropout imputation, the difference between the raw data and the imputed data in terms of value and distribution, shown in Fig. 3.

Figure 3a shows the number of dropout imputations generated by the eight methods on five scRNA-seq datasets. AGImpute achieves significantly fewer dropout imputations than other methods, and only 3.4% of the dropout imputations are generated by scImpute. Figure 3b provides the gene expression density plots of eight imputation methods on the Adam dataset. The dots in the plots represent cells, the *x*-axis indicates the mean expression level of all genes in the cell before imputation, and the *y*-axis indicates the value after imputation. It visualizes the distribution of cells and describes the difference between the raw data and the imputed data. To quantify the differences, the Pearson correlation coefficient is calculated. In the gene expression density plots of AGImpute, cells distribute along the 45° line showing a minimal difference from the raw data. In other methods, scattered distributions are exhibited which indicates a greater difference from the raw data. Moreover, the Pearson correlation coefficient also validates this finding. Therefore, AGImpute imputes the few dropout events while preserving more original information.

### 3.3 Comparing the clustering performance of different imputation methods

To evaluate the effect of dropout imputation on clustering performance, eight imputation methods are applied with hierarchical clustering on five scRNA-seq datasets.

**Figure 4.**
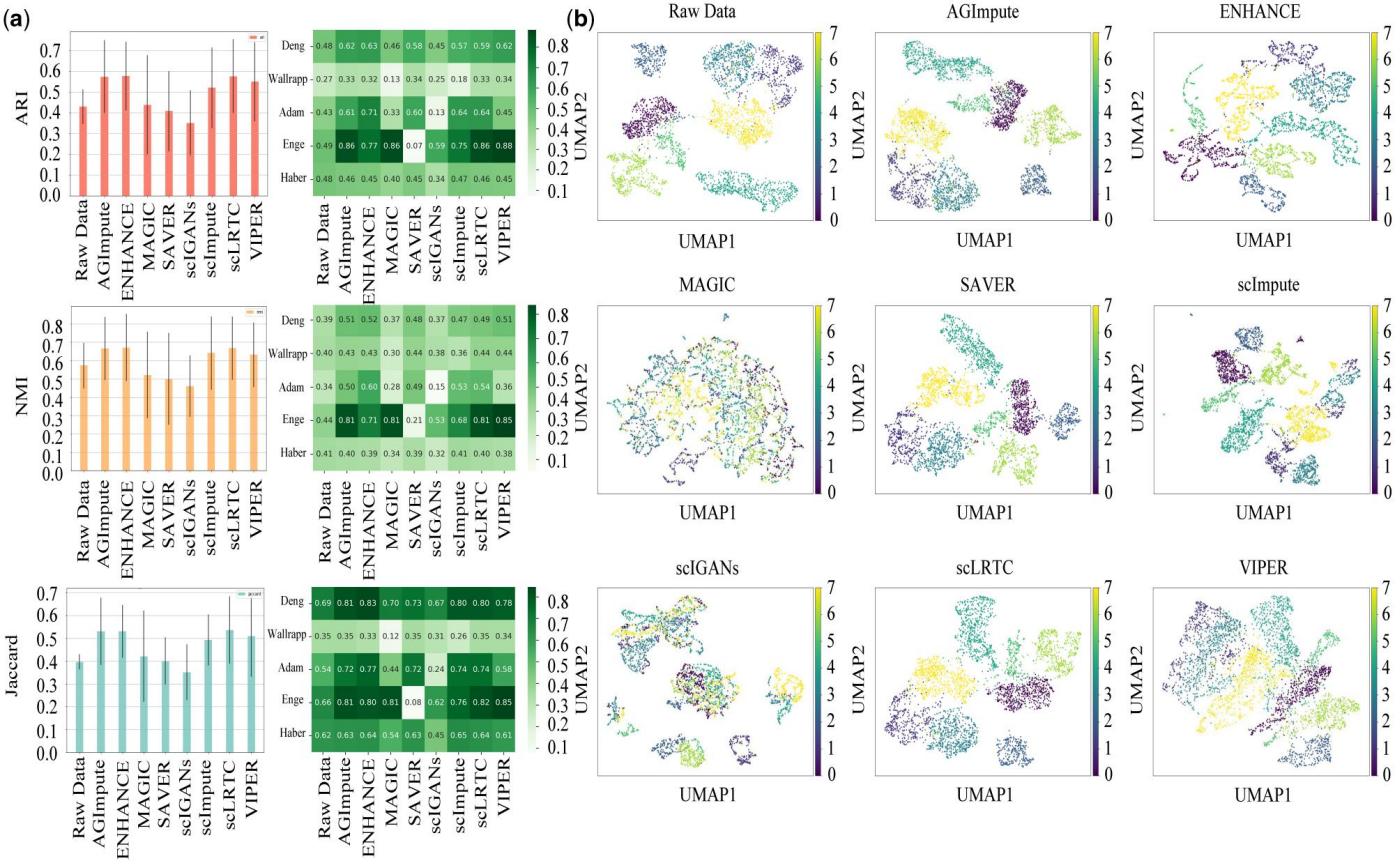
Evaluation of clustering performance with imputation methods on five scRNA-seq datasets. (a) ARI, NMI, and Jaccard index of clustering results. Bar graphs of mean and variance are presented in the left column, while a heatmap of clustering metrics is shown in the right column. (b) Visualization of the raw data and the imputed data by eight imputation methods on the Adam dataset using UMAP.

Clustering performance is evaluated in terms of ARI, NMI, and Jaccard index. A higher value closer to 1 indicates better clustering performance. The results are shown in Fig. 4a, in which bar graphs of mean and variance are presented in the left column, while a heatmap of clustering metrics is shown in the right column. In the left columns, AGImpute, ENHANCE, and scLRTC show the best clustering performance, while scIGANs performs poorly. In the right column, AGImpute maintains stable clustering performance on all five datasets. However, we find that AGImpute has no obvious advantage in clustering performance compared with ENHANCE and scLRTC. The reason is that AGImpute has little impact on clustering performance due to its small amount of imputation and small impact on data distribution. Further, to observe the cell type identification, Uniform Manifold Approximation and Projection (UMAP) is performed on the Adam dataset, which is shown in Fig. 4b. The results of AGImpute, SAVER, and scLRTC are similar to the raw data, while the other four methods confuse distinguishing cell types. Therefore, it is found that AGImpute improved clustering performance without altering the cell types of the raw data.

### 3.4 Analyzing the cell-specific marker genes

**Figure 5.**
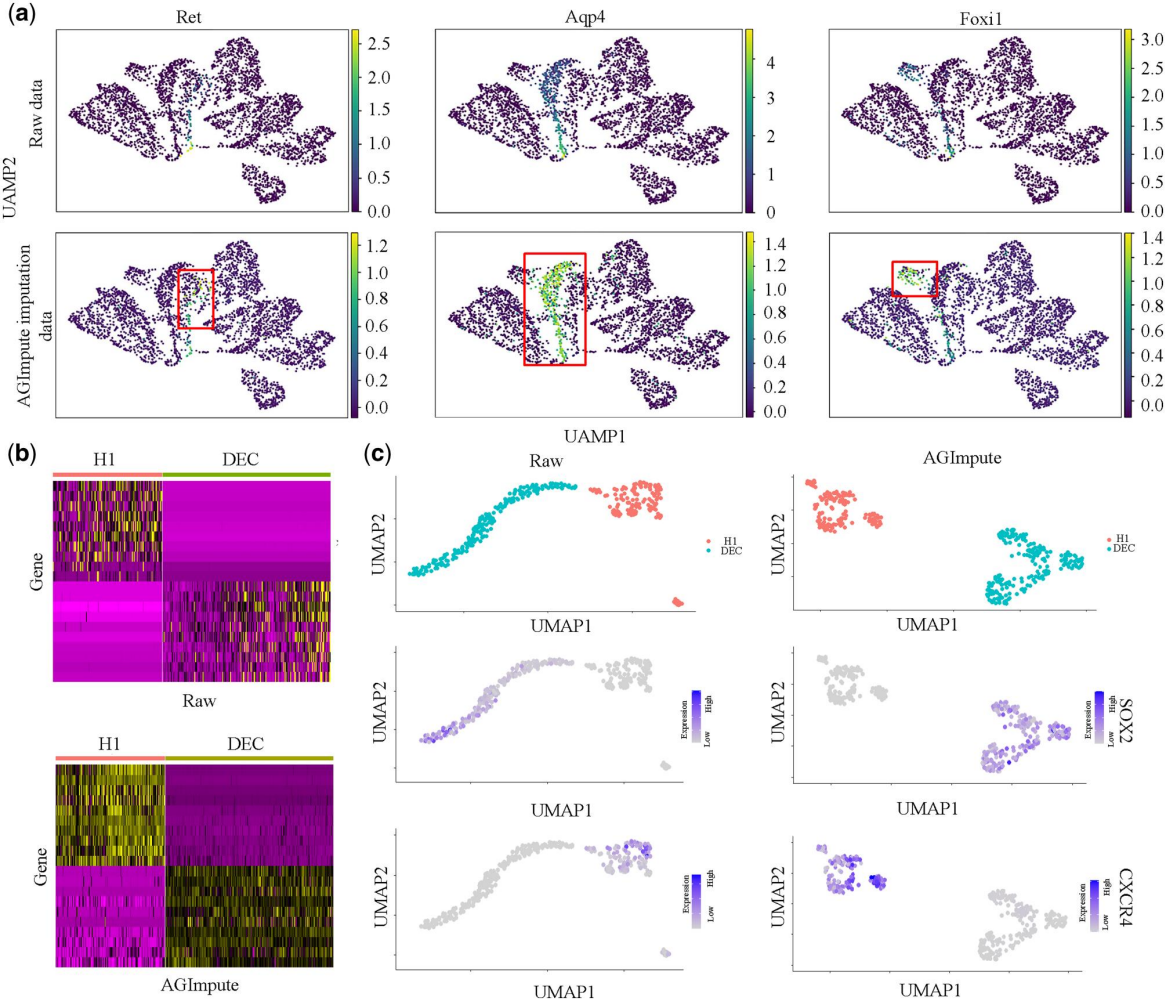
Evaluation of AGImpute-imputed data on the Cell Type dataset. (a) Visualization of the raw data and the AGImpute-imputed using UMAP. (b) Heatmap of gene expression in H1 and DEC subtypes. (c) Visualization of cell-specific marker genes, SOX2 and CXCR4, in the raw data and the AGImpute-imputed data.

The cell-specific marker genes of the raw data and AGImpute-imputed data on the Cell Type dataset are shown in Fig. 5, respectively.

Figure 5a provides the visualization results based on UMAP. We observe the expression changes in three cell-specific marker genes including Ret, Aqp4, and Foxi1, before and after imputation. The mutations or rearrangements of the Ret gene promote tumor formation, while the Aqp4 gene plays an essential role in brain water balance, astrocyte migration, nerve signal transmission, and neuroinflammation. The specific function of Foxi1 is unclear, but it may play an important role in cochleo-vestibular development and embryonic development. In the raw data, the expression of the Ret gene is low, the expression of the Aqp4 gene is relatively high, and the expression of the Foxi1 gene is moderate. In contrast, AGImpute-imputed data enhance the expression of the three genes significantly for improved cell type identification. Figure 5b shows the gene expression for H1 and DEC subtypes. The AGImpute-imputed data have a clearer block structure and more significant cell-specific marker genes than the raw data indicating AGImpute enhance these markers. Figure 5c provides the raw data and the AGImpute-imputed data with cell-specific marker genes of SOX2 and CXCR4. AGImpute-imputed data are more clearly divided into two types with cells in the same class more clustered and cells from different classes more separated. The gene SOX2 is highly expressed in the DEC subtype, while the gene CXCR4 is highly expressed in the H1 subtype. AGImpute improves cell type classification and enhances the differential expression of marker genes.

### 3.5 Inferencing trajectory

To understand cell development, trajectory inference organizes cells with time courses in different developmental stages (Herring *et al.*, 2017). Cell development trajectories are inferred by projecting each cluster onto a pseudo time point after cell clustering (Zhu *et al.*, 2023). We analyzed trajectory inference on the Time-course dataset, which differentiates human ES cells at known time points (0, 12, 24, 36, 72, and 96 h), shown in Supplementary Fig. S1a.

Supplementary Figure S1b visualizes the trajectory inference using Scanpy on the raw data and the AGImpute-imputed data. In the raw data, the developmental trajectories between 00, 12, and 22 h, as well as between 36 and 72 h are not inferred. AGImpute-imputed data recoveries the trajectories between 00–12 h, 12–24h and 36–72 h, but also shows incorrect trajectory association between 00 and 24 h. Overall, The AGImpute-imputed data outperform the raw data in trajectory inference.

## 4 Discussion

To differentially impute dropout events in different cells in scRNA-seq data, we have developed a method AGImpute. This tool includes a dropout identification module and a hybrid deep learning module. The dropout identification module differentially estimates the number of dropout events in different cells by dynamically estimating thresholds. The hybrid deep learning model combines Autoencoder with GAN to impute the identified dropout events based on integrating information from similar cells and gene expression distribution.

AGImpute provides three advantages over existing imputation methods for scRNA-seq data: (i) A mixed distribution combining ZIP, Gaussian, and ZINB is defined to fit scRNA-seq data better than the commonly used ZINB. ZINB is unsuitable for high-noise data, so it is only used to initialize the parameters of ZIP and the proportion of dropout events. (ii) The probability difference matrix is proposed to capture the changes in dropout probability and preserve gene expression heterogeneity. A regression strategy is used to accurately identify the number of dropout events for different cells. (iii) We precluster scRNA-seq data and perform the hybrid deep-learning module to train each cluster individually. Our approach converts the gene expression of individual cells into images by an encoder, which is input into a GAN to impute dropout events without overfitting.

AGImpute outperforms seven state-of-the-art methods in dropout events imputation and downstream analysis for scRNA-seq datasets, as demonstrated through the evaluation of two simulated datasets and seven scRNA-seq datasets. AGImpute adaptively identifies the fewest number of dropout events while preserving more original information, and it also improves the identification and visualization of cell type, enhances the cell-specific marker genes, and shows better clustering performance and trajectory inference. The experimental results provide compelling evidence of AGImpute’s superiority over the other imputation methods.

## Supplementary Material

btae068_Supplementary_Data

## References

[btae068-B1] Adam M , Potter AS, Potter SS. Psychrophilic proteases dramatically reduce single-cell RNA-seq artifacts: a molecular atlas of kidney development. Development 2017;144:3625–32. 28851704 10.1242/dev.151142 PMC5665481

[btae068-B2] Anderson JR , Morin EE, Brayer KJ et al Single-cell transcriptomic heterogeneity between conduit and resistance mesenteric arteries in rats. Physiol Genomics 2023;55:179–93. 36912534 10.1152/physiolgenomics.00126.2022 PMC10085562

[btae068-B3] Berrevoets J , Imrie F, Kyono T et al To impute or not to impute? Missing data in treatment effect estimation. In: *International Conference on Artificial Intelligence and Statistics*. Valencia, Spain: PMLR, 2023, 3568–90.

[btae068-B4] Buettner F , Natarajan KN, Casale FP et al Computational analysis of cell-to-cell heterogeneity in single-cell RNA-sequencing data reveals hidden subpopulations of cells. Nat Biotechnol 2015;33:155–60. 25599176 10.1038/nbt.3102

[btae068-B5] Chen L , Wang W, Zhai Y et al Single-cell transcriptome data clustering via multinomial modeling and adaptive fuzzy k-means algorithm. Front Genet 2020;11:295. 32362908 10.3389/fgene.2020.00295 PMC7180207

[btae068-B6] Chen M , Zhou X. VIPER: variability-preserving imputation for accurate gene expression recovery in single-cell RNA sequencing studies. Genome Biol 2018;19:1–15. 30419955 10.1186/s13059-018-1575-1 PMC6233584

[btae068-B7] Chu L-F , Leng N, Zhang J et al Single-cell RNA-seq reveals novel regulators of human embryonic stem cell differentiation to definitive endoderm. Genome Biol 2016;17:173. 27534536 10.1186/s13059-016-1033-x PMC4989499

[btae068-B8] Deng Q , Ramsköld D, Reinius B et al Single-cell RNA-seq reveals dynamic, random monoallelic gene expression in mammalian cells. Science 2014;343:193–6. 24408435 10.1126/science.1245316

[btae068-B9] Fanai H , Abbasimehr H. A novel combined approach based on deep Autoencoder and deep classifiers for credit card fraud detection. Expert Syst Appl 2023;217:119562.

[btae068-B10] Gan S , Deng H, Qiu Y et al DSAE-Impute: learning discriminative stacked autoencoders for imputing single-cell RNA-seq data. Curr Bioinform 2022;17:440–51.

[btae068-B11] Goodfellow I , Pouget-Abadie J, Mirza M et al Generative adversarial nets. In: *Advances in Neural Information Processing Systems*. Peachtree City, Georgia: MIT Press, 2014, 27.

[btae068-B12] Grün D , Kester L, Van Oudenaarden A. Validation of noise models for single-cell transcriptomics. Nat Methods 2014;11:637–40. 24747814 10.1038/nmeth.2930

[btae068-B13] Haber AL , Biton M, Rogel N et al A single-cell survey of the small intestinal epithelium. Nature 2017;551:333–9. 29144463 10.1038/nature24489 PMC6022292

[btae068-B14] Herring CA , Banerjee A, McKinley ET et al Unsupervised trajectory analysis of single-cell RNA-seq and imaging data reveals alternative tuft cell origins in the gut. Cell Syst 2017;6:37–51.e9. 29153838 10.1016/j.cels.2017.10.012 PMC5799016

[btae068-B15] Huang M , Wang J, Torre E et al SAVER: gene expression recovery for single-cell RNA sequencing. Nat Methods 2018;15:539–42. 29941873 10.1038/s41592-018-0033-z PMC6030502

[btae068-B16] Lee EY , Christiano AM, Dai Z et al 065 Longitudinal analysis of T cell dynamics in alopecia areata at single-cell resolution. J Investig Dermatol 2022;142:S11.

[btae068-B17] Li WV , Li JJ. An accurate and robust imputation method scImpute for single-cell RNA-seq data. Nat Commun 2018;9:997. 29520097 10.1038/s41467-018-03405-7 PMC5843666

[btae068-B18] Mahmoudi MR. Cyclic clustering approach to impute missing values for cyclostationary hydrological time series. Qual Quant 2023;57:2619–39.

[btae068-B19] Miao Z , Li J, Zhang X. scRecover: discriminating true and false zeros in single-cell RNA-seq data for imputation. bioRxiv, 2019, 665323, preprint: not peer reviewed. 10.1101/665323. 39912305

[btae068-B20] Pan X , Li Z, Qin S et al ScLRTC: imputation for single-cell RNA-seq data via low-rank tensor completion. BMC Genomics 2021;22:860. 34844559 10.1186/s12864-021-08101-3 PMC8628418

[btae068-B21] Qiu Y , Yan C, Zhao P et al SSNMDI: a novel joint learning model of semi-supervised non-negative matrix factorization and data imputation for clustering of single-cell RNA-seq data. Brief Bioinform 2023;24:bbad149. 37122068 10.1093/bib/bbad149

[btae068-B22] Raevskiy M , Yanvarev V, Jung S et al Epi-Impute: single-cell RNA-seq imputation via integration with single-cell ATAC-seq. Int J Mol Sci 2023;24:6229. 37047200 10.3390/ijms24076229 PMC10094055

[btae068-B23] Song Z , Henze L, Casar C et al Human γδ T Cell Identification from Single-Cell RNA Sequencing Datasets by Modular TCR Expression. Oxford, United Kingdom: Oxford University Press, 2023. 10.1093/jleuko/qiad069 37437101

[btae068-B24] Vallejos CA , Risso D, Scialdone A et al Normalizing single-cell RNA sequencing data: challenges and opportunities. Nat Methods 2017;14:565–71. 28504683 10.1038/nmeth.4292 PMC5549838

[btae068-B25] van Dijk D , Sharma R, Nainys J et al Recovering gene interactions from single-cell data using data diffusion. Cell 2018;174:716–29.e27. 29961576 10.1016/j.cell.2018.05.061 PMC6771278

[btae068-B26] Wagner F , Barkley D, Yanai I. ENHANCE: accurate denoising of single-cell RNA-seq data. bioRxiv, 2019, 1–9, preprint: not peer reviewed. 10.1101/655365.

[btae068-B27] Wallrapp A , Riesenfeld SJ, Burkett PR et al The neuropeptide NMU amplifies ILC2-driven allergic lung inflammation. Nature 2017;549:351–6. 28902842 10.1038/nature24029 PMC5746044

[btae068-B28] Wang J , Huang M, Torre E et al Gene expression distribution deconvolution in single-cell RNA sequencing. Proc Natl Acad Sci USA 2018;115:E6437–46. 29946020 10.1073/pnas.1721085115 PMC6048536

[btae068-B29] Xu Y , Zhang Z, You L et al scIGANs: single-cell RNA-seq imputation using generative adversarial networks. Nucleic Acids Res 2020;48:e85. 32588900 10.1093/nar/gkaa506 PMC7470961

[btae068-B30] Zappia L , Phipson B, Oshlack A. Splatter: simulation of single-cell RNA sequencing data. Genome Biol 2017;18:174. 28899397 10.1186/s13059-017-1305-0 PMC5596896

[btae068-B31] Zhu H , Wang G, Nguyen-Ngoc K-V et al Understanding cell fate acquisition in stem-cell-derived pancreatic islets using single-cell multiome-inferred regulomes. Dev Cell 2023;58:727–43.e11. 37040771 10.1016/j.devcel.2023.03.011 PMC10175223

