# Supplementary material for: AGImpute: imputation of scRNA-seq data based on a hybrid GAN with dropouts identification
Source: Bioinformatics. 2024 Feb 5;40(2):btae068. doi: 10.1093/bioinformatics/btae068 (PMC10877090; doi:10.1093/bioinformatics/btae068)
Supplement: btae068_Supplementary_Data [file btae068_supplementary_data.zip › Final-SupplementaryFile.docx]

Inferencing trajectory


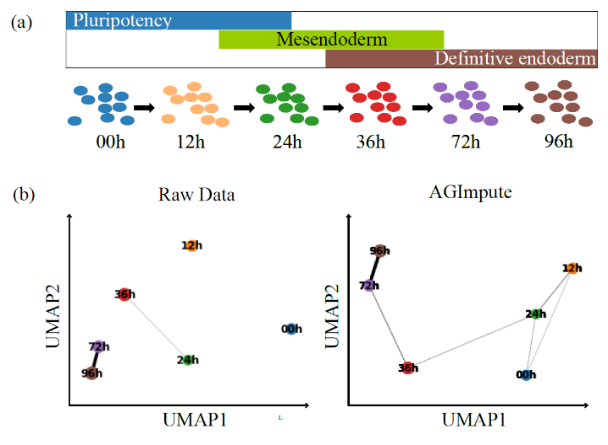


Fig. S1 Improvement of trajectory inference with AGImpute. (a) Time-course dataset; (b) Trajectory inference with the raw data and the AGImpute-imputed data.

Comparing the performance between AGImpute and scRecover

Different from AGImpute, scRecover fits scRNA-seq data based on ZINB distribution and predicts the number of true zeros based on the species accumulation curve. AGImpute is compared to scRecover on the CellType dataset. The evaluation metrics include the number of dropout imputations, ARI, NMI, and the cell types visualization by UMAP, shown in Fig.S2. In the literature, scRecover is combined with scImpute to imputate the dropouts. So, scImpute is used to compare the performance between AGImpute and scRecover.

Fig.S2(a) shows the number of dropout imputations. In the scRecover-scImpute method, scRecover is used to identify the dropouts, and scImpute is used to impute the dropouts. AGidentify is the dropout identification part of AGImpute. In the AGidentify-scImpute method, AGidentify is used to identify the dropouts, and scImpute is used to impute the dropouts. From the results of Fig. S2(a), the number of imputations for scRecover is slightly less than that of AGidentify.

Fig.S2(b) shows the ARI, NMI, and cell types identification by UMAP. Compared with the raw data, the ARI of scImpute is slightly improved, and the NMI is reduced. Both ARI and NMI are reduced for scRecover-scImpute. Both ARI and NMI are improved for AGidentify-scImpute. Therefore, the AGidentify method improves the data quality while the number of imputations is much lower than that of scImpute. AGidentify obtains higher clustering performance than scRecover. Finally, it can be observed that AGidentify-scImpute achieves the clearest classification of cell types.

Analyzing the influence of selecting different values of threshold *t*

In the dynamic threshold regression mechanism of AGImpute, a parameter *t* is defined to adjust the number of dropouts. This parameter is defined as the number of dropouts whose confidence is credible, and the number of the top *t* dropouts is used as the basis for adjusting the number of dropouts in each cell. Based on the CellType dataset, we analyze the influence of selecting different *t* values in terms of the number of imputations, ARI, and NMI, shown in Fig. S3.

Fig.S3(a) shows the number of dropout imputations using different *t* values. It can be seen that the number of imputations does not change when the value of *t* is 20 to 160. As the value of *t* increases, the number of imputations decreases linearly.

Fig.S3(b) also shows the ARI, NMI, and cell types visualization by UMAP. It can be seen that ARI and NMI are higher and more stable for smaller *t*. The best ARI, NMI is obtained when *t* is 20. So, we set *t* to 20 as the default value.


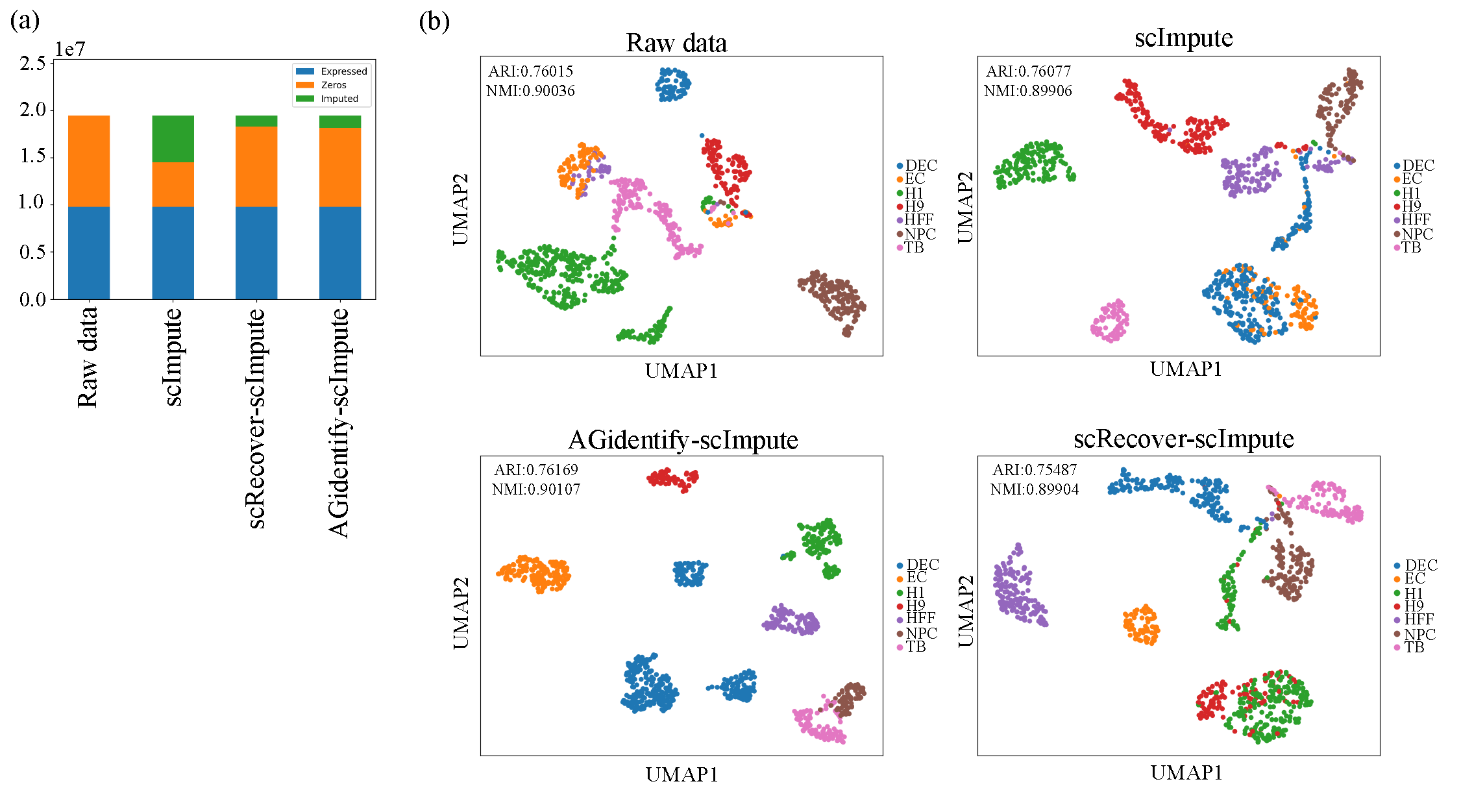


Fig. S2 Performance of AGImpute and scRecover. (a) The number of imputations; (b) ARI, NMI, and cell types visualization.


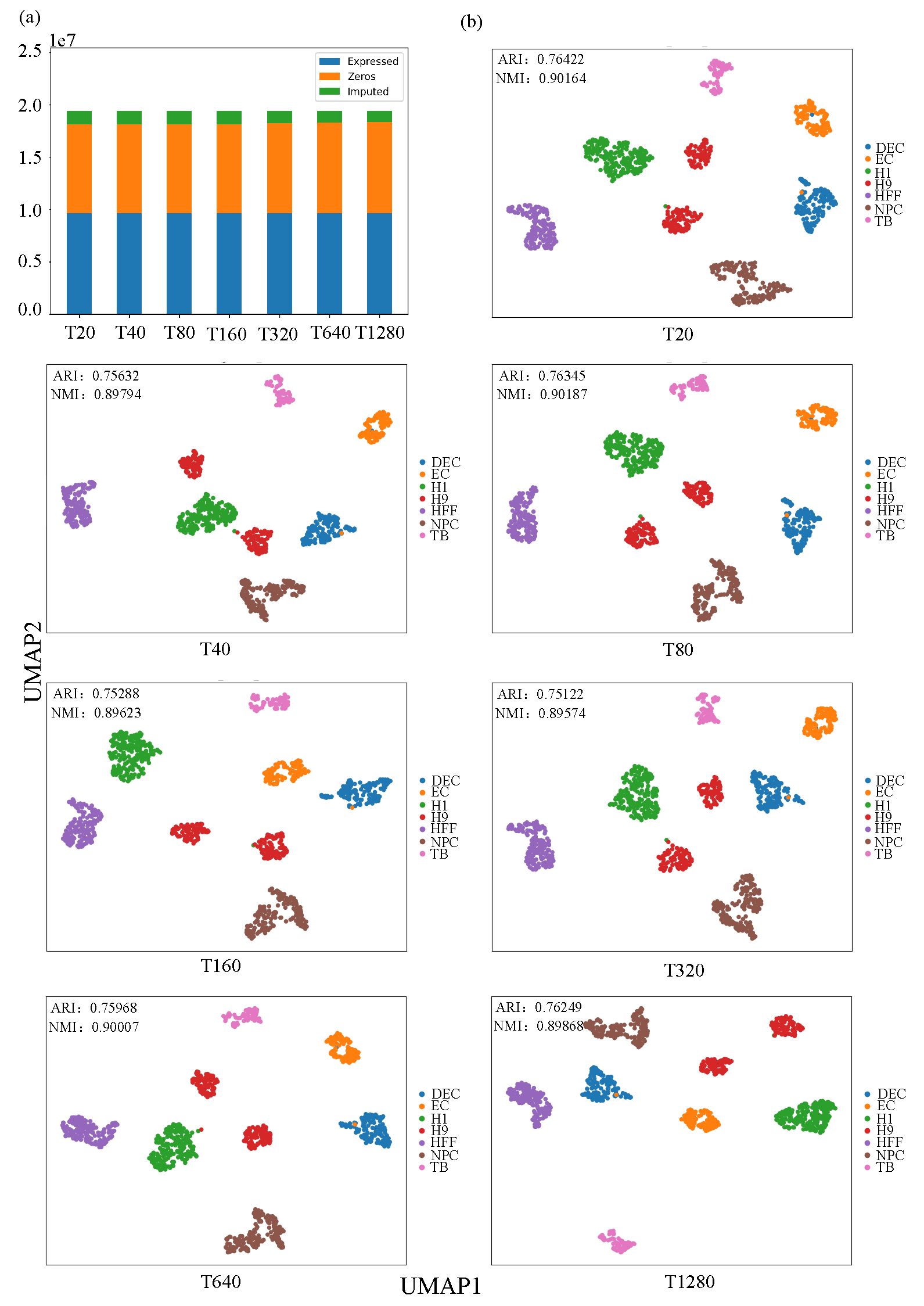


Fig. S3 The performance with different *t* values. (a) The number of imputations; (b) ARI, NMI, and cell types visualization.
