# Supplementary figures and images for: AGImpute: imputation of scRNA-seq data based on a hybrid GAN with dropouts identification
Source: Bioinformatics. 2024 Feb 5;40(2):btae068. doi: 10.1093/bioinformatics/btae068 (PMC10877090; doi:10.1093/bioinformatics/btae068)

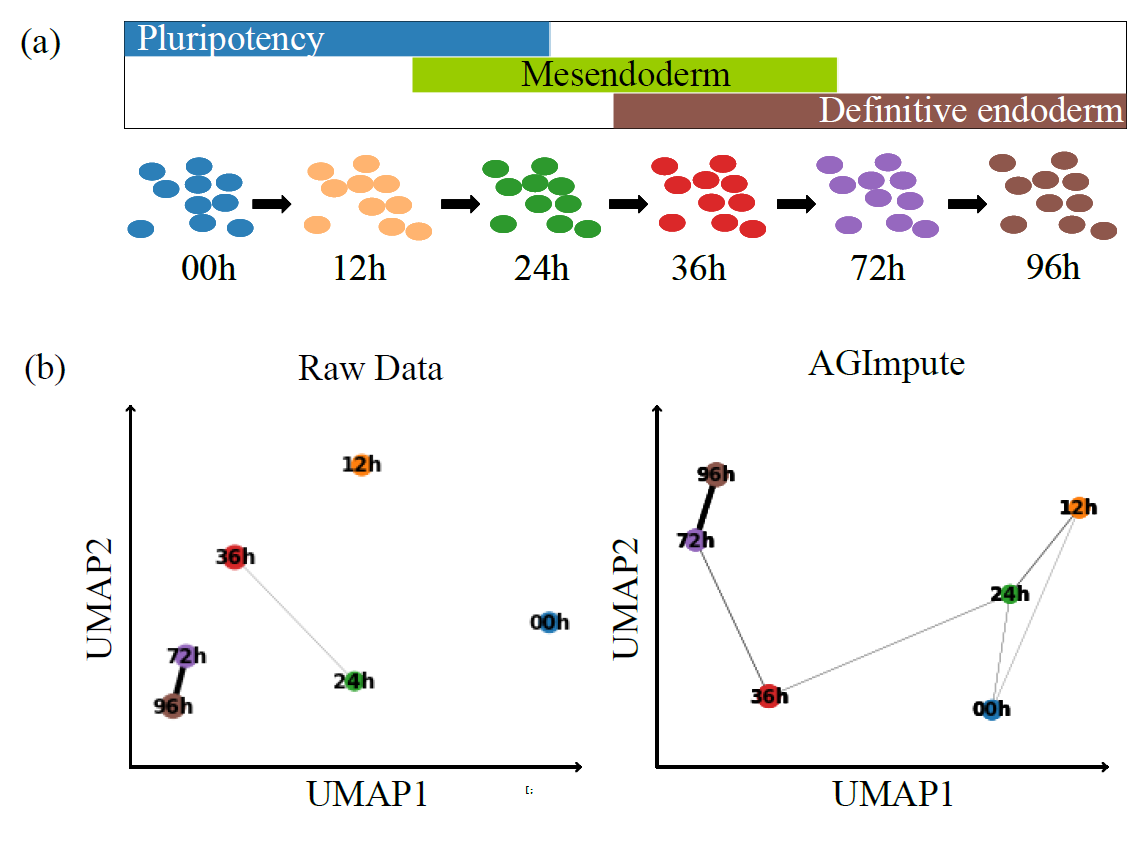

Supplement: btae068_Supplementary_Data [file btae068_supplementary_data.zip › Fig.S1.png]

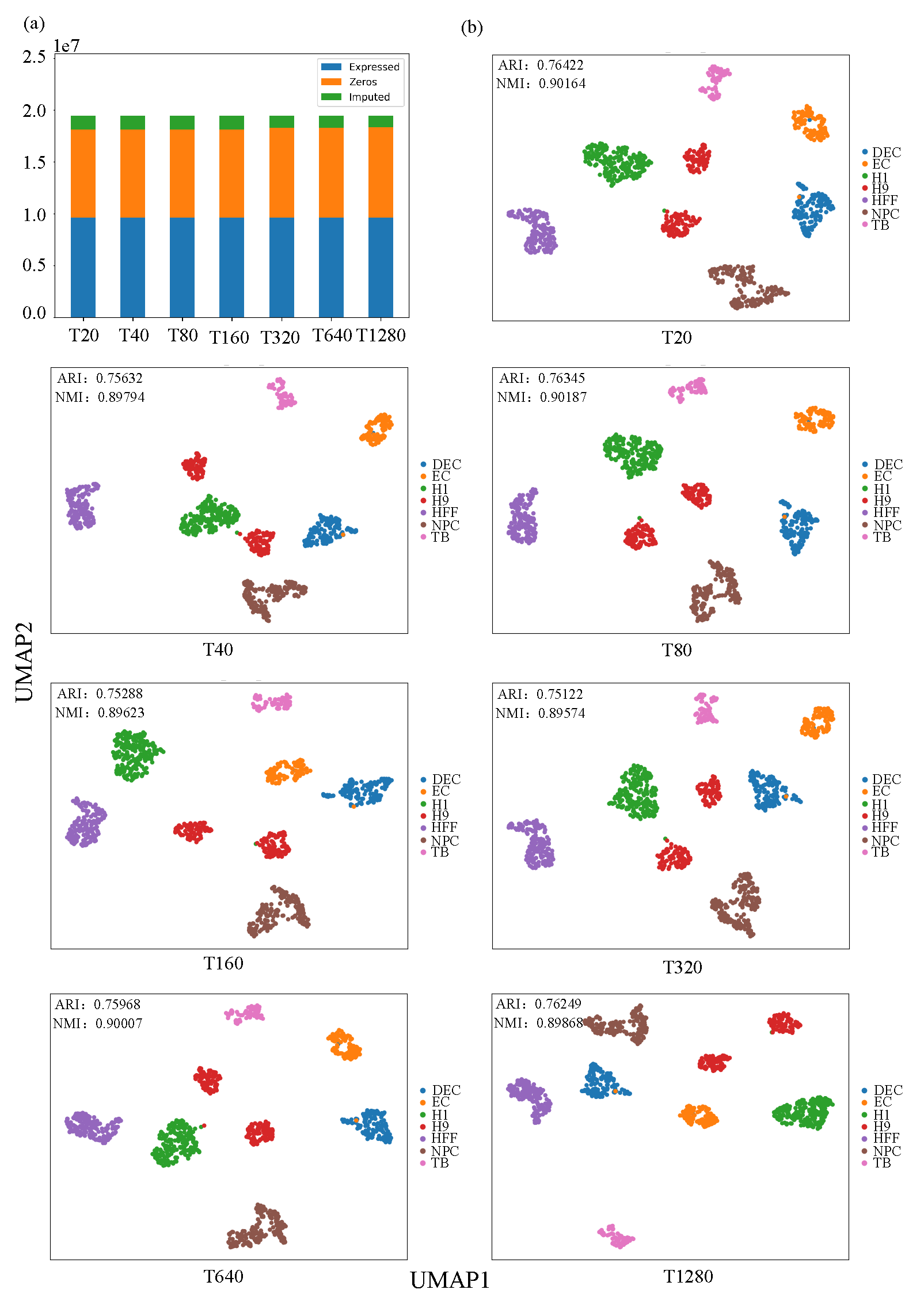

Supplement: btae068_Supplementary_Data [file btae068_supplementary_data.zip › Fig.S3.png]

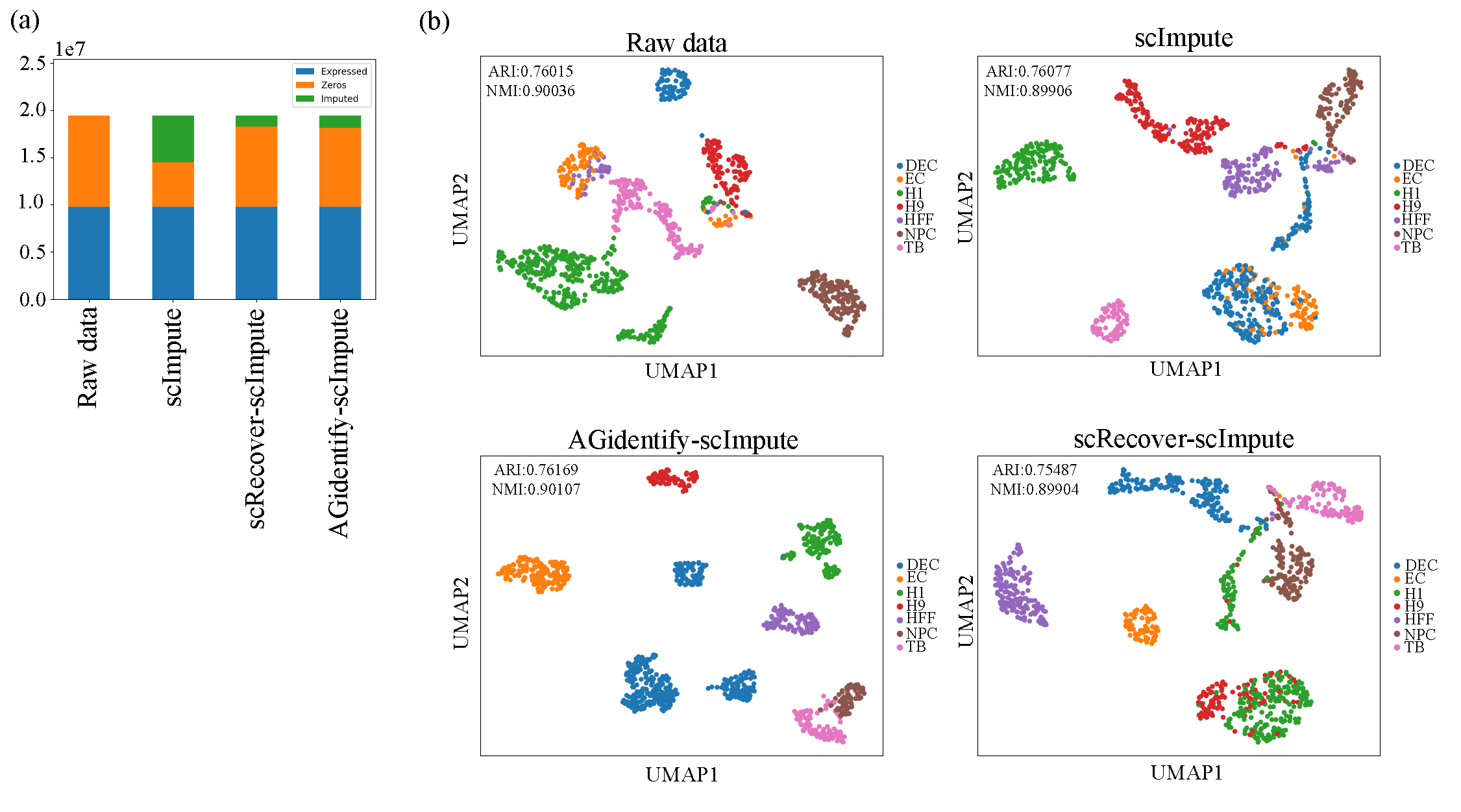

Supplement: btae068_Supplementary_Data [file btae068_supplementary_data.zip › Fig.S2.png]
